# Stringent Response Regulates Stress Resistance in Cyanobacterium *Microcystis aeruginosa*

**DOI:** 10.3389/fmicb.2020.511801

**Published:** 2020-11-12

**Authors:** Hui Jin, Yong Min Lao, Ke Zhen Ying, Jin Zhou, Zhong Hua Cai

**Affiliations:** ^1^Shenzhen International Graduate School, Tsinghua University, Shenzhen, China; ^2^School of Pharmaceutical Sciences (Shenzhen), Sun Yat-sen University, Guangzhou, China; ^3^Institute for Advanced Study, Shenzhen University, Shenzhen, China

**Keywords:** stringent response, stress tolerance, *Microcystis aeruginosa*, cyanobacterial blooms, guanosine 3′,5′-bisdiphosphate

## Abstract

Cyanobacterial blooms are serious environmental issues in global freshwater ecosystems. Nitrogen limitation is one of the most important strategies to control cyanobacterial blooms. However, recent researches showed that N limitation does not effectively control the bloom; oppositely, N limitation induces N-fixing cyanobacterial blooms. The mechanism underlying this ecological event is elusive. In this study, we found that N limitation enhances stress tolerance of *Microcystis aeruginosa* by triggering stringent response (SR), one of the most important bacterial adaptive responses to environmental stresses. Initiation of SR exerted protective effects on the cells against salt and oxidative stresses by promoting colony formation, maintaining membrane integrity, increasing photosynthetic performance, reducing ROS production, upregulating stress-related genes, etc. These protections possibly help *M. aeruginosa* maintain their population number during seasonal N limitation. As SR has been proven to be involved in nitrogen fixing under N limitation conditions, the potential role of SR in driving the shift and succession of cyanobacterial blooms was discussed. Our findings provide cellular evidence and possible mechanisms that reducing N input is ineffective for bloom control.

## Introduction

Cyanobacterial blooms are global environmental issues that threaten the freshwater ecosystem (Paerl et al., 2011). Cyanobacteria produce taste and toxic compounds and therefore endanger the safety of drinking water, poison zooplankton, and fish and even cause the death of humans through the food chain (Szlag et al., 2015). Large accumulation of cyanobacteria induces anoxia and causes the death of aquatic organisms (Paerl and Tucker, 1995). At the end of the bloom, dead cyanobacteria and fish floating on the surface contaminate water and destroy the landscape. Cyanobacterial blooms pose serious threats to public health and lead to a lot of undesirable economic, ecological, and resource problems (Havens, 2008). Despite decades of research and billions of dollars spent on it, the problem of cyanobacterial blooms persists and appears to have worsened in some regions.

To control cyanobacterial blooms, many possible causes have been studied and proposed. The emphasis has been heavily on curtailing the input of nutrients, including N, P, Fe, and Si (Paerl et al., 2011; Molot et al., 2014). In addition, temperature, salinity, light, and climate changes are also considered to be related to cyanobacterial blooms (Paerl et al., 2001; Yang et al., 2016). Although restriction of P input has been implemented widely since the 1960s, the need to reduce N input for controlling cyanobacterial blooms was proposed in recent years (Conley et al., 2009; Paerl et al., 2011). However, recent studies showed that reducing N input flavored N-fixing cyanobacteria and therefore could not effectively control cyanobacterial blooms (Schindler et al., 2008; Lewis, 2017; Higgins et al., 2018). The mechanism behind this ecological event is elusive. As early habitants on Earth, cyanobacteria acquired complex strategies to survive various environmental stresses during their long evolutionary history (Kulasooriya, 2011). For example, cyanobacteria that cannot fix nitrogen are able to survive prolonged periods of nitrogen starvation as chlorotic cells in a dormant state (Klotz et al., 2016). Dormant-like cyanobacterial cells can resist environmental stresses at a larger extent than normal vegetable cells. Therefore, we cannot gauge the true effect of N limitation on cyanobacterial blooms without thoroughly understanding the stress tolerance mechanisms underlying the ability of cyanobacteria to survive in prolonged N starvation. Given the observed increase in N-limited and N&P co-limited freshwater systems worldwide (Elser et al., 2007; Moisander et al., 2009), we hypothesize that stress tolerance activated by N limitation could help non-N-fixing cyanobacterial cells survive more easily than other planktonic algae. But the stress tolerance mechanism activated by N limitation is unknown in many cyanobacteria.

To survive in N deprivation conditions, cyanobacteria have evolved several adaptative mechanisms. Stringent response (SR), a global regulatory system, is activated by N deprivation and leads to metabolic change from fast growth into survival maintenance (Masuda, 2012). SR is mediated by an effector molecule, guanosine 3′,5′-bisdiphosphate (ppGpp), which is derived from guanosine pentaphosphate (pppGpp) by hydrolysis. Collectively, pppGpp and ppGpp are termed (p)ppGpp (Braeken et al., 2006). A growing number of studies indicate that SR regulates many biological processes in bacteria, such as growth adaption (Potrykus et al., 2011), antibiotic production (Eymann et al., 2001; Inaoka and Ochi, 2002), morphological differentiation (Dahl et al., 2005), sporulation (Chakraburtty and Bibb, 1997; Hesketh et al., 2007), social behavior (Van Delden et al., 2001), and fruit body development (Harris et al., 1998). In *Escherichia coli*, two proteins are involved in SR: RelA and SpoT. RelA is associated with ribosomes and produces ppGpp in response to amino acid starvation (Wendrich et al., 2002). SpoT is a bifunctional enzyme which exhibits strong degradative and little synthetic activities; it regulates ppGpp level under most stresses (Gentry and Cashel, 1996; Magnusson et al., 2005).

SR plays an important role in defending abiotic stress, such as nutrient deprivation (Vinella et al., 2005; Rifat et al., 2009; Nguyen et al., 2011), temperature (Yang and Ishiguro, 2003), salt stress (Takahashi et al., 2004), and biotic stress including pathogen infection (van der Biezen et al., 2000). In most bacteria, SR lies at the top of the network to govern global gene expression in response to environmental stress. During stress response, SR not only stimulates expression of stress-induced genes but also activates transcription of genes that mediate general defenses (Traxler et al., 2011). General defense activated by SR confers cyanobacteria resistance to environmental stress. For instance, in *E. coli*, upon exposure to nutrient depletion, SR upregulated genes involved in RopS-dependent response, a general acclimation response, and carbon catabolism genes for cells to switch to alternative carbon source (Traxler et al., 2006, 2008, 2011). Also, SR enhanced tolerance to antibiotics by curtailing production of prooxidant metabolites in *Pseudomonas aeruginosa* (Nguyen et al., 2011).

*Microcystis aeruginosa*, one of the most common non-N-fixing cyanobacteria in bloom, has long been a primary focus. In the course of evolution, *M. aeruginosa* acquired sophisticated strategies to improve stress tolerance, for example, dormant-like cell formation for surviving prolonged period of nutrient-limited conditions (Zou et al., 2018), colony formation for protecting cells from stress (Yang et al., 2005), and buoyancy regulation for overcoming the vertical separation of light and nutrients in the water column (Thomas and Walsby, 1985). In this study, we endeavor to (1) identify the SR system, the key *Marsh* gene, in *M. aeruginosa*; (2) determine the tolerance ability of *M. aeruginosa* to environmental stress conferred by SR; (3) disclose possible mechanisms underlying the SR-induced stress tolerance; and (4) discuss the role of SR in cyanobacterial blooms.

## Materials and Methods

### Bacterial Strains and Growth Conditions

*M. aeruginosa* NIES-843 strain was obtained from the Institute of Hydrobiology, Chinese Academy of Sciences. *E. coli* strains CF1648 and CF1693 were kindly donated by Michael Cashel (Gentry and Cashel, 1996). *M. aeruginosa* cells were cultivated in conical flasks containing 100 ml of Blue-Green medium (BG-11) in an illumination incubator at 25°C for constant illumination (20 μmol photons m^–2^ s^–1^ of cool white fluorescent lights) under a 12 h light/12 h dark cycle and shaken manually twice daily.

#### Triggering SR in *M. aeruginosa*

To trigger SR, *M. aeruginosa* cells were exposed to classic stresses, such as nitrogen depletion, hyperosmotic stress, oxidative stress, and serine hydroxamate (SHX), which is a classic inducer of SR. Then the transcription of *Marsh* and the ppGpp level were determined to evaluate whether SR was initiated. For N depletion, *M. aeruginosa* cells grown to the logarithmic phase were washed three times with N-deficient BG-11 (without nitrate and sodium nitrate). Then cells were cultivated in conical flasks containing 100 ml of N-deficient BG-11 for different times (0, 0.5, 1, 2, 4, 6, 8, 10, 12, 24, 48, and 72 h). For hyperosmotic stress, NaCl was added to the culture at a final concentration of 20 mg/ml (2%, mass fraction) and maintained for different times (0, 0.5, 1, 2, 4, and 6 h). For oxidative stress, H_2_O_2_ was added to the culture at a final concentration of 0.2 mg/ml (0.02%, mass fraction) for different times (0, 1, 2, 4, 6, 8, and 10 h). For the induction of SHX, SHX was added to the culture at a final concentration of 1 g/L and maintained for different times (0, 15, 30, 60, 90, and 120 min).

#### Stress Tolerance

To test whether the initiation of SR enhances the tolerance of *M. aeruginosa* against environmental stresses, the algal cells were pretreated by N depletion or SHX for 2 h to trigger SR and then transferred into different stress conditions. The indicators, such as growth, cell integrity, and ROS, were used to evaluate the protective capability of SR. For hyperosmotic stress, SR-triggering cells by N depletion or SHX were transferred into BG-11 containing 0, 0.5, 1.0, 1.5, 2.0, 2.5, 3.0, 3.5, 4.0, and 4.5% NaCl (mass fraction unless otherwise specified). As control, logarithmic cells were transferred to BG-11 culture with the same concentration of NaCl. For oxidative stress, SR-triggering cells by N depletion or SHX were transferred into BG-11 containing 0, 0.01, 0.02, 0.03, 0.04, 0.05, 0.06, and 0.07% H_2_O_2_, respectively. As control, logarithmic cells were transferred to BG-11 culture with the same concentration of H_2_O_2_.

### Functional Complementation of Marsh in *E. coli*

3T3TTo identifying the system of SR, the key *Marsh* gene encoding the synthase and/or hydrolase domains for ppGpp synthesis and hydrolysis in *M. aeruginosa*, was cloned and characterized by functional complementation. The complemental analysis was based on the function restoration of *E. coli* mutants by transforming the *Marsh* gene. The *E. coli* wild-type strain CF1648 and its derived mutants, CF1652 (*relA*^–^ *spoT*^+^) and CF1693 (*relA*^–^ *spoT*^–^), were used for the complement analysis. Due to genetic modification, the CF1693 strain cannot grow on MM agar medium, while CF1652 is unable to grow on MM plate supplemented with amino acids serine, methionine, and glycine (SMG plate). As a positive control, CF1648 can grow on MM and SMG plates. All *E. coli* strains are able to grow on nutrient-rich medium, such as LB. To test whether MaRSH functions as an RSH, the growth of mutants CF1652 and CF1693 transformed with MaRSH on LB, MM, and SMG was examined. The open reading frame (ORF) of *Marsh* was amplified by PCR using genomic DNA as a template and then cloned into pET-32a-c(+) between *Bam*HI and *Hin*dIII sites, which generated plasmid pET-32a-*Marsh*. The plasmid 3T3TpET-32a-*Marsh* was transformed into CF1652 and CF1693. As a control, pET-32a-c(+) empty vector was also transformed into CF1652 and CF1693. The transformed strains were coated to LB, SMG, and MM plates and grown at 37°C for 24 h. Primers used in this study are listed in Supplementary Table S1.

### Protein Purification and Enzyme Assay

Recombinant MaRSH was produced in *E. coli* BL-21 containing the plasmid pET-32a-*Marsh*. Cells were pelleted by centrifugation at 12,000 × *g* for 2 min at 4°C. Enzyme was prepared by using the Ni-NTA Spin Kit (QIAGEN, Germany) according to the user protocol and eluted using 300 μl of PBS (50 mM NaH_2_PO_4_, 300 mM NaCl, pH 7.0) containing 500 mM imidazole. The PBS buffer was then changed to Tris-HCl buffer (0.1 M Tris-HCl, pH 8.0, 5 mM DTT, 1 mM EDTA) by using the Zeba Spin Desalting Columns (Thermo Fisher Scientific, United States). Protein extracts were quantitated at 25°C using the TaKaRa BCA Protein Assay Kit (TaKaRa, China).

The synthase and hydrolase activities of MaRSH were assayed according to previous reports (Nanamiya et al., 2008; Ito et al., 2012). Briefly, for synthase activity assay, reaction mixture containing 50 μM GDP and 80 μM ATP was incubated with 1 μg of recombinant MaRSH at 37°C for 20 min in the presence of 5 mM Mg^2+^. For hydrolase activity, reaction mixture containing 50 mM Tris-HCl (pH 8.0), 5 mM Mg^2+^, 50 μM ppGpp, and 1 μg of recombinant MaRSH was incubated at 37°C for 20 min. The mixtures were analyzed by UPLC on a HILIC chromatographic column. ppGpp and GDP products were detected by UV absorbance at 252 nm.

### Maximum Fluorescence Yield

ChI fluorescence was detected using Phyto-PAM (Walz, Germany). Initial (*F*_0_) and maximum (*F*_*m*_) fluorescence were measured after the cells had been incubated in darkness for 10 min. The maximum effective quantum yield of PSII electron transport was calculated as *F*_*v*_/*F*_*m*_ = *F*_0_/(*F*_*m*_ − *F*_0_).

### Laser Scanning Confocal Microscopy (LSCM) Analysis of Reactive Oxygen Species (ROS)

LSCM was performed according to Owusu-Ansah et al. (2008). The fluorescence of DC-FDA was excited with argon laser at 488 nm and detected at 530 nm, and autofluorescence of chloroplast was observed simultaneously at 680 nm.

### Detection of ROS

Intracellular ROS in cells were detected using 2′,7′-dichlorodihydrofluorescein diacetate (H_2_DCFDA) as a fluorescent probe according to Owusu-Ansah et al. (2008). In brief, *M. aeruginosa* cells were harvested and washed three times by PBS. Then, the cells were resuspended in PBS containing 100 μM H_2_DCFDA. The suspension was incubated at 30°C in the dark for 30 min and shaken every 10 min. Then, the mixture was washed again by PBS and resuspended in 400 μl of PBS. The fluorescence intensity was measured with the BD FACSCalibur^TM^ platform (BD Bioscience, United States), with excitation and emission filters of 485 and 530 nm, respectively.

### Quantitative RT-PCR (qRT-PCR)

To determine the expression levels of *Marsh* and genes encoding antioxidant enzymes such as SOD, POD, GAPX, and GR in *M. aeruginosa*, fresh cells were collected by centrifugation at 10,000 rpm for 10 min at 4°C. Total RNA was extracted from ∼10^7^ cells by using the TRIzol^®^ reagent (Thermo Fisher Scientific, United States). qRT-PCR was run on the 7300 Real-Time PCR system (Applied Biosystems, United States) using the PrimeScript^®^ RT reagent kit with gDNA eraser and the SYBR^®^ Premix Ex Taq^TM^ II Kit (TaKaRa, China). The reaction mixture contained 4 μl of cDNA, 0.5 μl of forward and reverse primer mix (20 μM each), 1 μl of 50 × ROX reference dye, and 25 μl of 2 × TaKaRa SYBR Green PCR mix in a final volume of 50 μl. All reactions were setup in triplicate and every sample was replicated in parallel three times to ensure statistical relevance. The qRT-PCR conditions were as follows: 30 s at 95°C, 40 cycles of 30 s at 95°C and 34 s at 60°C. Primer specificity was confirmed by RT-PCR amplification, which produced single amplicons with the expected size for each primer set; these amplicons were sequenced to validate their authenticity. Specificity of qRT-PCR was monitored by the presence of dissociation curves with single peaks. Data were analyzed using the SDS software (Applied Biosystems, United States). Primers used in this assay are listed in Supplementary Table S1.

### Extraction, Purification, and Detection of ppGpp

To determine the intercellular level of ppGpp in *M. aeruginosa* under different stress conditions, 2.4-g fresh cell weight (FCW) of *M. aeruginosa* cells was collected, frozen in liquid nitrogen, crushed, and resuspended in 10 ml of 1 M cool formic acid. The broken cells were vigorously mixed, incubated on ice for 30 min, and then centrifuged at 10,000 rpm for 10 min at 4°C. The upper layer was transferred to a new tube and centrifuged to remove cell debris at 10,000 rpm for 10 min at 4°C. The aqueous layer was then collected and purified by solid phase extract (SPE). SPE purification was performed by using the OASIS^®^ WAX 3-cc Vac Cartridges (Waters, United States) according to the manufacturer’s instructions. Chromatographic separation was performed on a Waters BEH amide column (2.1 × 150 mm, 1.7 μm) according to our published method (Jin et al., 2018).

### Extraction and Analysis of Exocellular Polymeric Substances (EPS)

Polysaccharides, proteins, and humic acids are the main components of the EPS matrix in *M. aeruginosa* (Sheng et al., 2010; Xu et al., 2014). Therefore, total EPS was measured as the sum of these three components. Cells were centrifuged at 8,000 × *g* for 15 min at 25°C. The pellets were resuspended in 10 ml of 0.05% NaCl (pH 10.0), heated at 45°C for 4 h, and centrifuged at 12,000 × *g* for 20 min at 4°C. The supernatant was collected for subsequent measurement. Polysaccharide content was measured using the anthrone–sulfuric acid method, with glucose as a standard (Laurentin and Edwards, 2003). Proteins and humic acids were determined according to Frølund (Frølund et al., 1996).

### Activity Assays of Antioxidant Enzymes

To detect the activities of antioxidant enzymes under different stress conditions, *M. aeruginosa* cells were harvested by centrifugation at 2,000 × *g* for 10 min at 25°C, washed with PBS buffer, and collected by centrifugation. The cells were resuspended with PBS buffer, sonicated, and centrifuged at 15,000 × *g* for 20 min at 4°C. The supernatant was used for activity assays. The activities of peroxidase (MaPOD), glutathione peroxidase (MaGAPX), superoxide dismutase (MaSOD), and glutathione reductase (MaGR) in *M. aeruginosa* were assayed using POD and GAPX, SOD, and GR detection kits (Beyone, China) according to the user protocol.

### Sequence Analysis

Sequence blast was performed using BLAST software^1^. Multiple alignments were conducted using Clustal X2.1. Secondary domains were predicted by Conserved Domain Search^2^. Homology modeling was performed by the SWISS-MODEL server^3^. Sequences used for multiple alignments are listed in Supplementary Table S2.

### Statistical Analysis

The data were processed by one-way analysis of variance using SPSS version 13.0 (SPSS, United States). Summary statistics were expressed as means ± SD.

## Results

### Characterization of the SR System in *M. aeruginosa*

**FIGURE 1:**
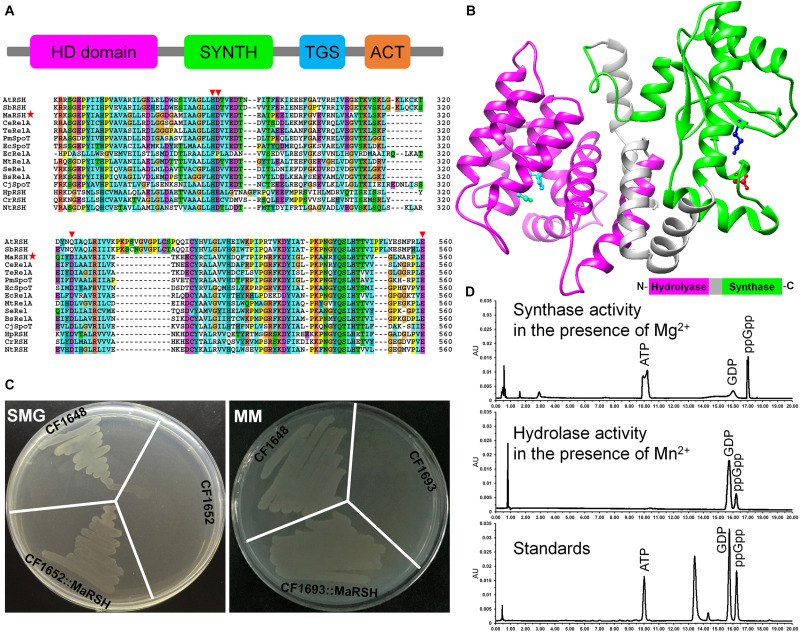
Characterization of *relA*/*spoT* homologous gene *Marsh* in *M. aeruginosa*. **(A)** Secondary conserved domains and sequence alignment of MaRSH. Inverted triangles indicate key regulatory amino acid residues. Red stars indicated the sequence of MaRSH. **(B)** Homology modeling of MaRSH. Key regulatory amino residues are indicated by ball-and-stick model, e.g., H_9__9_D_100_ of the hydrolase domain in cyan, D_287_ of the synthase domain in red, and E_347_ for metal binding in mazarine. **(C)** Functional complementation of MaRSH. **(D)** *In vitro* activities of MaRSH. In the presence of Mg^2+^, the enzyme utilized ATP and GDP as substrates to synthesize ppGpp (upper panel), while in the presence of Mg^2+^, MaRSH displayed hydrolase activity to hydrolyze ppGpp into GDP (lower panel).

Although the homolog of *relA*/*spoT* is annotated in the genome of *M. aeruginosa* NIES-843 (NC_010296.1), at present, its authentic function is unknown. Therefore, the sequence, designated as MaRSH according to its dual functions, was isolated and functionally characterized. Using *M. aeruginosa* genomic DNA as template, the ORF of *Marsh* was amplified, which encodes a polypeptide with 765 aa in length. This MaRSH shows a high level of similarity to the RelA/SpoT superfamily from eubacteria and plants (Figure 1A). It has typical secondary structures of the RelA/SpoT superfamily, e.g., HD domain (hydrolase), RelA_SpoT domain (synthase), TGS domain, and ACT domain. Using homology modeling, the protein structure of MaRSH was constructed. The hydrolase and synthase domains were mapped onto the model using *Streptococcus equisimilis* Rel (PDB ID: 1vj7) as a template (Figure 1B). These results implied that MaRSH might be a RelA/SpoT-like enzyme.

The function of MaRSH was determined by functional complementation in *E. coli* strains CF1652 (*relAspoT*^–^), CF1693 (*relA*^–^*spoT*^–^), and CF1648 (wild type). All these *E. coli* strains could grow on an LB plate (data not shown). CF1652 could not grow on an SMG plate, but CF1652 transformed with pET-32a-MaRSH restored the growth on an SMG plate (Figure 1C). Meanwhile, as a RelA/SpoT double-mutant strain, CF1693 could not grow on the MM plate (Xiao et al., 1991). As shown in Figure 1C, CF1693 transformed with the pET-32a-c(+) empty vector could not grow on an MM plate, while transforming pET-32a-MaRSH into CF1693 could restore the growth on an MM plate (Figure 1C). These results indicated that MaRSH could complement both *relA*^–^*spoT*^+^ and *relA*^–^*spoT*^–^ phenotypes and function as an RSH, i.e., as a (p)ppGpp synthase and hydrolase bifunctional enzyme. The *in vitro* activities of MaRSH were determined (Figure 1D). In the presence of Mn^2+^, MaRSH hydrolyzed ppGpp into GDP. When Mg^2+^ was present, MaRSH synthesized ppGpp using ATP and GDP as substrates. These results finally indicated that (1) MaRSH is a bifunctional (p)ppGpp synthetase/guanosine-3′,5′-bis(diphosphate) 3′-pyrophosphohydrolase; (2) the synthase activity of MaRSH is Mg^2+^ dependent while the hydrolase activity is Mn^2+^ dependent; and (3) sophisticated regulation between hydrolase and synthase activities implies a complex regulation of SR in stress response.

### N Depletion Triggers SR in *M. aeruginosa*

**FIGURE 2:**
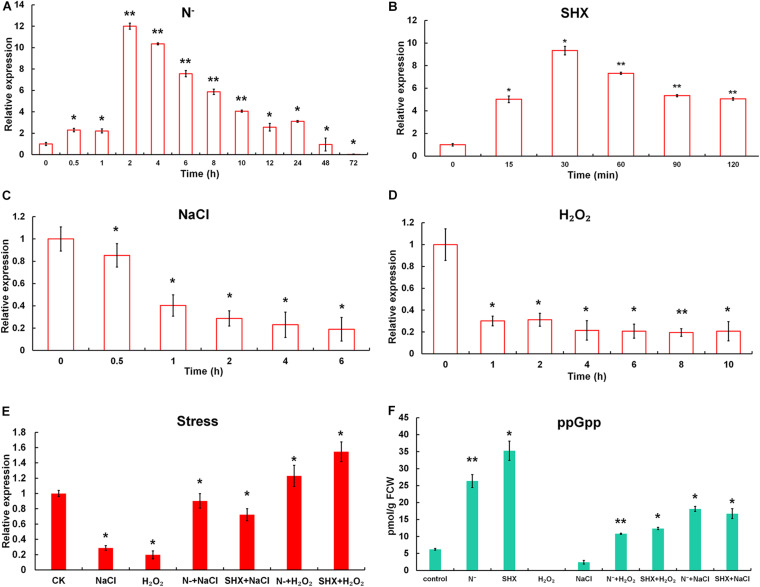
N depletion triggered SR in *M. aeruginosa*. **(A–D)** Time course of *Marsh* expression under N depletion (N^–^), SHX, NaCl, and H_2_O_2_. **(E)** Transcription of *Marsh* in NaCl- and H_2_O_2_-stressed cells pretreated with N depletion (N^–^ + NaCl and N^–^ + H_2_O_2_) or SHX (SHX + NaCl and SHX + H_2_O_2_). **(F)** Endogenous ppGpp synthesized under stresses. Error bars indicate the standard deviation (SD) of the mean (*n* = 3) for *Marsh* transcription analysis or *n* = 6 for endogenous ppGpp determination. *p < 0.05; **p < 0.01.

The effect of N depletion, hyperosmotic stress, oxidative stress, and the classic SR inducer SHX on the transcription of *Marsh* was determined (Figure 2). When cells were transferred to N-deficient BG-11 (N^–^), the transcription of *Marsh* was rapidly upregulated to more than twofold within 30 min (*p* < 0.05) and reached its maximum (about 12-fold) at 2 h (*p* < 0.01), compared to the control. Similarly, the transcriptional expression of *Marsh* was induced to the highest level (almost 10-fold higher than the control group) by SHX after 30 min (*p* < 0.05). In contrast, *Marsh* was inhibited by NaCl and H_2_O_2_. It is worth noting that the inhibition could be reversed by pretreatment with N depletion (N^–^ + NaCl and N^–^ + H_2_O_2_) or SHX (SHX + NaCl and SHX + H_2_O_2_). When cells were exposed to 2.0% NaCl or 0.04% H_2_O_2_ for 2 h, *Marsh* was downregulated to 28.7 or 31.2% of the control, respectively. While in cells pretreated with N depletion or SHX, the transcription of *Marsh* under the corresponding conditions was restored to near normal levels (*p* < 0.05) or was obviously (*p* < 0.05) upregulated.

To further clarify whether *M. aeruginosa* triggered SR in response to N depletion, the content of ppGpp in cells was determined. As shown in Figure 2F, in cells subjected to N depletion for 2 h or SHX for 30 min, *in vivo* ppGpp significantly increased to 26.33 ± 1.91 pmol/g FCW (*p* < 0.01) or 35.29 ± 2.84 pmol/g FCW (*p* < 0.05), compared to the control (6.24 ± 0.25 pmol/g FCW). By contrast, high salinity led to a marked (*p* < 0.05) decrease in ppGpp (2.42 ± 0.55 pmol/g FCW); no ppGpp was detected in cells treated by oxidative stress. However, pretreatment with N depletion or SHX reversed the inhibitory effect; i.e., ppGpp increased to 10.81 ± 0.18 (N^–^ + H_2_O_2_), 11.26 ± 0.21 (SHX + H_2_O_2_), 18.13 ± 0.72 (N^–^ + NaCl), and 16.74 ± 1.44 pmol/g FCW (SHX + NaCl). These results indicated that (1) both N depletion and SHX initiated SR; (2) as a typical SR inducer, SHX could be used as a positive control; (3) hyperosmotic and oxidative stresses inhibited SR in *M. aeruginosa*; and (4) pretreatment with N depletion or SHX compensated SR inhibition by hyperosmotic or oxidative stress.

### SR Maintains Membrane Integrity to Alleviate Cell Viability Inhibition by Environmental Stress

**FIGURE 3:**
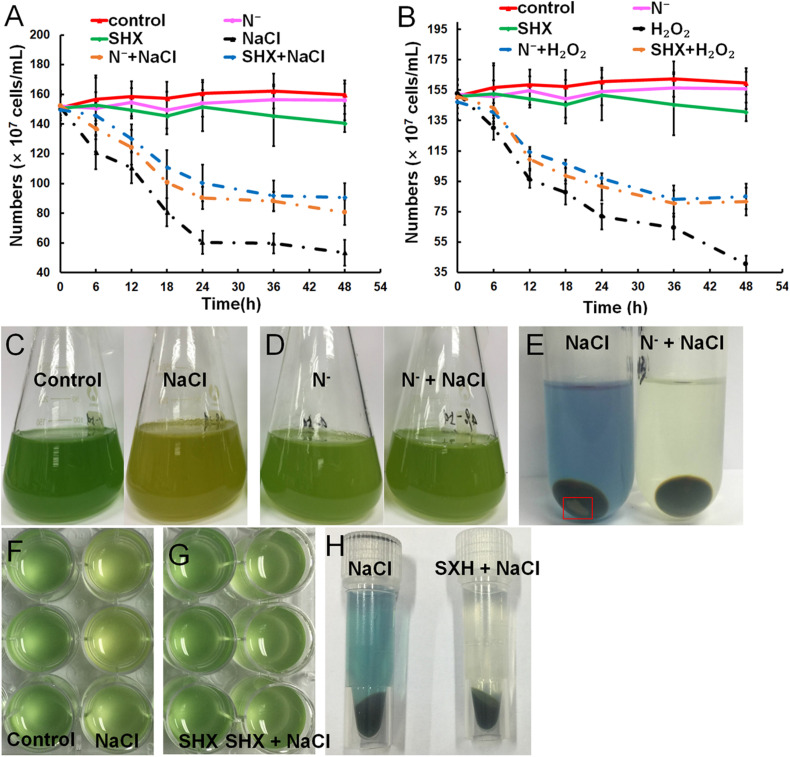
Relief of stress-induced growth inhibition by SR through maintaining membrane integrity. **(A,B)** Growth curves of *M. aeruginosa* cells under stresses. **(C–H)** *M. aeruginosa* cell cultures in stress conditions. Error bars indicate the standard deviation (SD) of the mean (*n* = 6).

Gradual cell growth arrest was observed in cells exposed to hyperosmotic or oxidative stress over time, whereas N depletion or SHX had little influence on the growth (Figures 3A,B). Pretreatment with N depletion for 2 h or SHX for 30 min significantly (*p* < 0.05) alleviated cell growth arrest induced by hyperosmotic or oxidative stress. Hyperosmolarity induced by high salinity can destroy cell membrane to inhibit cell growth (Zhu, 2016). Therefore, the growth inhibition by salt stress is likely to be related to membrane damage. Visually, high salinity changed the color of cell culture from blue-greenish to brown-yellowish (Figures 3C,F), while pretreatment with N depletion (Figure 3D) or SHX (Figure 3G) had no visual change. Moreover, the supernatant of NaCl-treated cells showed blue, and the precipitate contained some debris in the bottom of the tube (Figures 3E,H), indicating that membrane permeability increased, and some cells were lysed under salt stress. Whereas pretreatment with N depletion or SHX had little effect on the cells, as the supernatant of cells was colorless, no cell debris was found in the precipitate (Figures 3E,H).

### SR Promotes Cluster Formation in *M. aeruginosa* Against Environmental Stress

**FIGURE 4:**
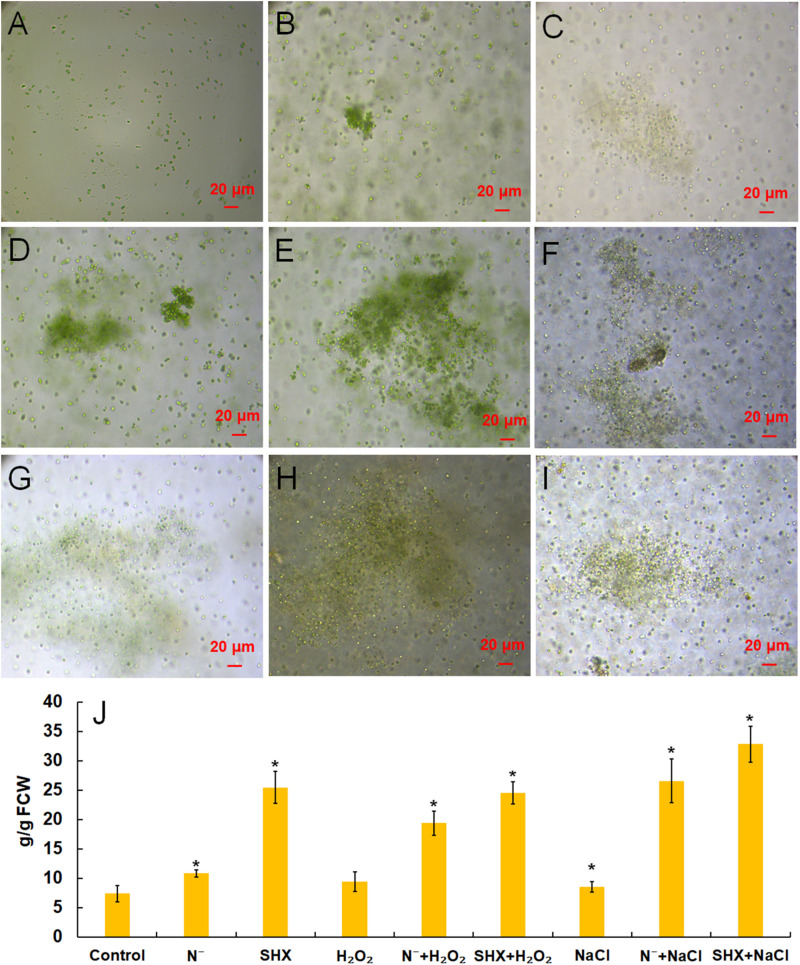
SR promoted the formation of cell clusters and EPS production. **(A–I)** Control, NaCl, H_2_O_2_, N depletion (N^–^), SHX, N^–^ + NaCl, N^–^ + H_2_O_2_, SHX + NaCl, and SHX + H_2_O_2_. **(J)** Contents of EPS under stresses. Error bars indicate the standard deviation (SD) of the mean (*n* = 6). *p < 0.05.

*M. aeruginosa* only exists as a single cell or a few paired cells in laboratory culture. To be exact, it lost the ability to cluster in the laboratory (Figure 4A). However, the cluster could be reconstituted by NaCl (Figure 4B), H_2_O_2_ (Figure 4C), N depletion (Figure 4D), and SHX (Figure 4E), which was accompanied by production of EPS, i.e., 8.53 ± 0.87 (*p* < 0.05), 9.42 ± 1.64 (*p* < 0.05), 10.80 ± 0.60 (*p* < 0.05), and 25.41 ± 2.72 mg/g FCW (*p* < 0.05), respectively, compared to the control (7.36 ± 0.42 mg/g FCW) (Figure 4J). Pretreatment with N depletion or SHX enlarged the size of the cluster and further (*p* < 0.05) enhanced EPS production to 26.57 ± 3.72 (N^–^ + NaCl, Figure 4F), 19.36 ± 2.04 (N^–^ + H_2_O_2_, Figure 4G), 32.81 ± 3.05 (SHX + NaCl, Figure 4H), and 24.52 ± 1.92 mg/g FCW (SHX + H_2_O_2_, Figure 4I). These results suggested that SR promoted cluster formation by enhancing EPS production to withstand environmental stress.

### SR Maintains Photosynthetic Performance Under Stress

**FIGURE 5:**
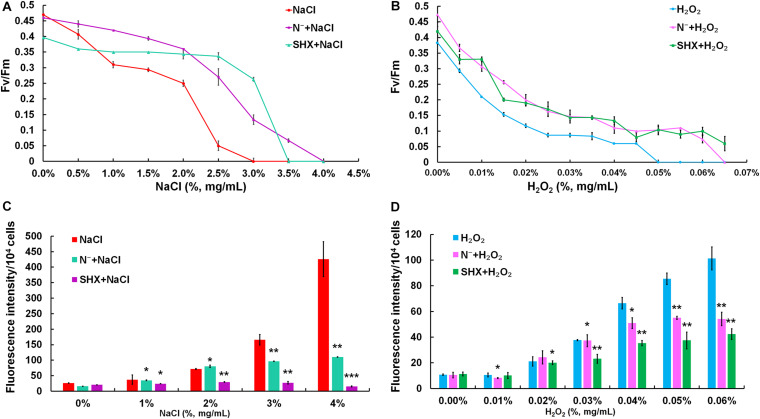
SR maintained photosynthetic performance and reduced ROS production. **(A,B)** Maximum quantum yield (F_*v*_/F_*m*_) induced by stresses. **(C,D)** Dose-dependent ROS levels in *M. aeruginosa* under stresses. Error bars indicate the standard deviation (SD) of the mean (*n* = 6). *p < 0.05; **p < 0.01; ***p < 0.001.

The photochemical reaction efficiency of PSII, *F*_*v*_/*F*_*m*_, is commonly used to determine metabolic disorder of cells subjected to environmental stress, which describes the state of photodamage (Baker, 2008). As shown in Figures 5A,B, although the *F*_*v*_/*F*_*m*_ ratio in all treatment groups showed a decreasing trend along with the increasing exposure concentrations, the *F*_*v*_/*F*_*m*_ ratios of SR-induced cells (N^–^ + NaCl, SHX + NaCl, N^–^ + H_2_O_2_, and SHX + H_2_O_2_) were higher than those of non-SR-induced cells (NaCl and H_2_O_2_) at every corresponding concentration. Furthermore, in the groups of N^–^ + NaCl and SHX + NaCl, the F_*v*_/F_*m*_ ratios declined to zero at NaCl concentrations of 4.0 and 3.5%, respectively, while those of NaCl-treated cells declined to zero at 3.0%. In other words, when SR was triggered by N depletion or SHX, the salt tolerance of *M. aeruginosa* (the minimum salt concentration leading to the reduction of the *F*_*v*_/*F*_*m*_ ratio to zero) increased at 1.0 and 0.5%, respectively. A similar result was observed in H_2_O_2_-treated cells: N^–^ + H_2_O_2_ and SHX + H_2_O_2_ at 0.065 and 0.066%, respectively, and H_2_O_2_ at 0.05%.

### SR Reduces Oxidative Damage by Environmental Stress

**FIGURE 6:**
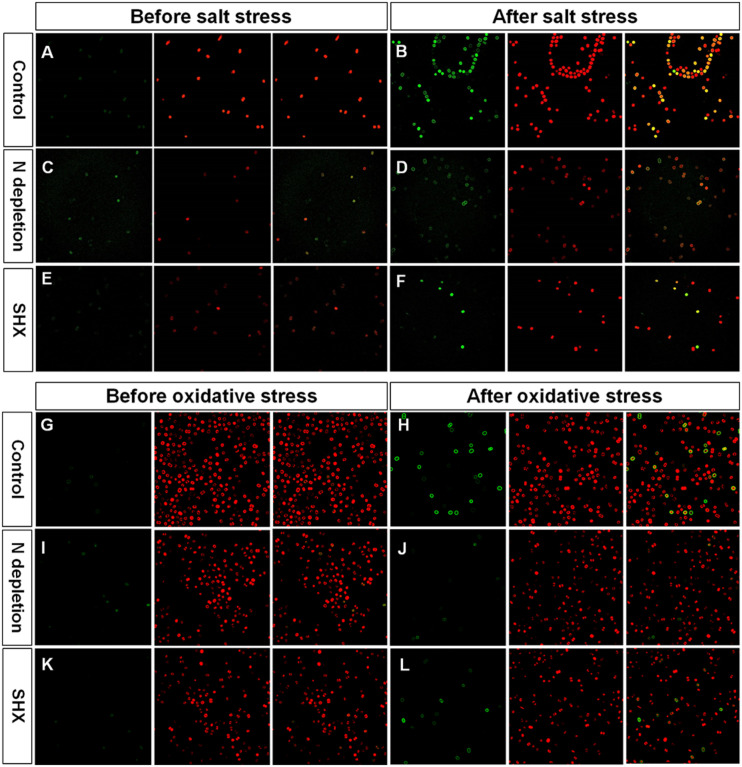
Fluorescence of ROS and chlorophyll in *M. aeruginosa* cells under stresses. **(A–L)** Control, NaCl, N depletion (N^–^), SHX, N^–^ + NaCl, N^–^ + H_2_O_2_, SHX + NaCl, and SHX + H_2_O_2_. Left panels: ROS fluorescence (green). Middle panels: Chlorophyll autofluorescence (red). Right panels: Merged images of ROS fluorescence and chlorophyll autofluorescence (yellow).

Environmental stress disrupts cellular homeostasis and leads to the generation of ROS (Bowler et al., 1992; Polle, 2001). ROS attacks cellular macromolecules, such as proteins, nucleic acids, and membrane lipids. Therefore, ROS is commonly used as a reliable indicator to evaluate cell damage by stress. As depicted in Figures 5C,D, the level of ROS in NaCl- or H_2_O_2_-treated cells soared with the rising concentrations and reached about 10-fold of the control at 4% NaCl or 0.06% H_2_O_2_. Although the fluorescent intensities of ROS in SR-induced cells (N^–^ + NaCl, N^–^ + H_2_O_2_, and SHX + H_2_O_2_) also increased with the increasing exposure concentrations, the levels were significantly (*p* < 0.05 or 0.01) lower than those in non-SR-induced cells (NaCl and H_2_O_2_) at every corresponding concentration. The intracellular ROS was visualized by ROS fluorescence. Before NaCl or H_2_O_2_ stressing, the ROS signal was low in SR-induced cells (N^–^ and SHX) and the control (Figures 6A,C,E,G,I,K). After stressing, the ROS signal increased remarkably in non-SR-induced cells (Figures 6B,H), while in SR-induced cells, the ROS signal slightly strengthened and was far below that in non-SR-induced cells (Figures 6D,F,J,L). These data suggested that SR inhibited ROS formation induced by stress.

### SR Upregulates Stress-Related Enzymes

**FIGURE 7:**
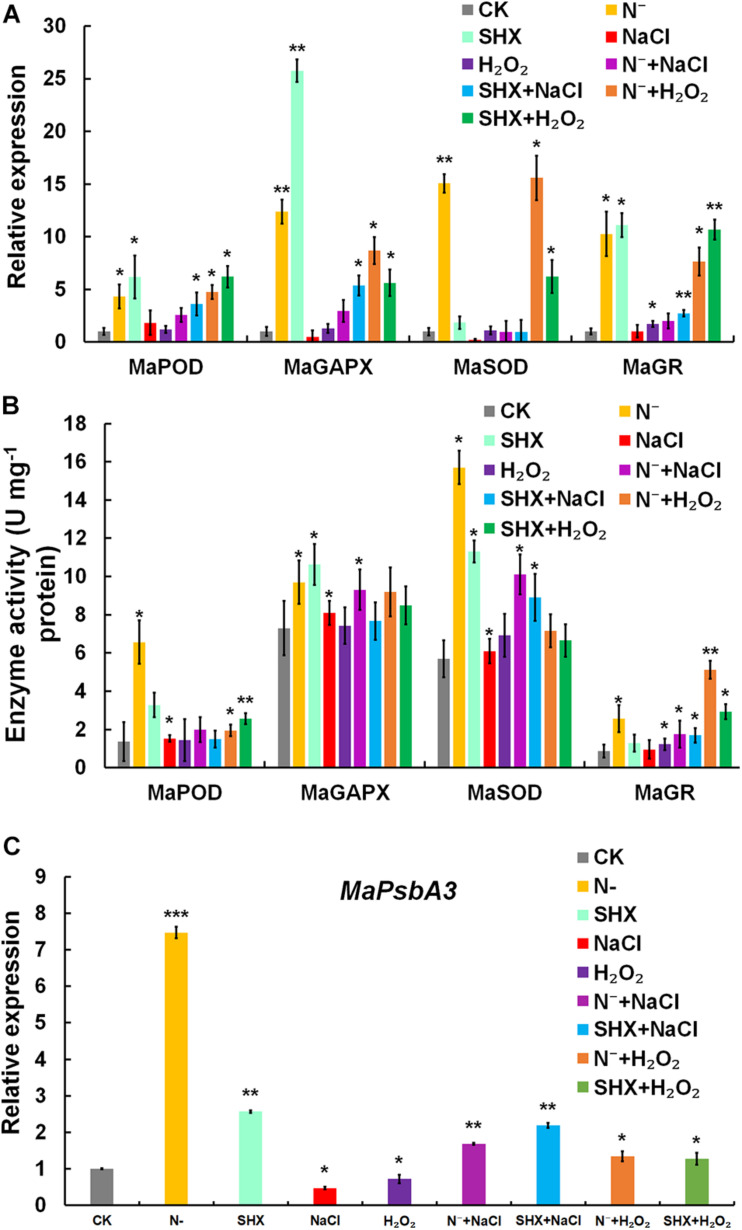
SR enhanced ROS elimination of ROS by upregulating the expression of stress-related genes. **(A)** Transcription of stress-related genes under stresses. **(B)** Enzyme activity of stress-related genes under stresses. **(C)** Transcription of D1-encoding *MaPsbA3* under stresses. Error bars indicate the standard deviation (SD) of the mean (*n* = 3). *p < 0.05; **p < 0.01; ***p < 0.001.

Transcription of typical antioxidant enzymes, such as MaPOD, MaGAPX, MaSOD, and MaGR, was determined by qRT-PCR. MaPOD, MaGAPX, MaSOD, and MaGR were induced by N depletion or SHX (Figure 7A). When these SR-induced cells were subjected to stresses (N^–^ + NaCl, SHX + NaCl, N^–^ + H_2_O_2_, and SHX + H_2_O_2_), the upregulation was maintained, though lower than that of N^–^ and SHX. Generally, transcription of these antioxidant enzymes in SR-induced cells was higher than that in non-SR-induced cells (NaCl and H_2_O_2_). *In vitro* activity of these enzymes was also determined. An overall correlation of activity to transcription was observed (Figure 7B).

In PSII, a dynamic cycle of photodamage and photorepair is achieved by the degradation and resynthesis of D1, which binds all redox-active components in electron transfer. Abnormal turnover of D1 could lead to ROS burst by hindering electron transfer. D1 is regulated by SR at the transcriptional level in cyanobacteria and plants (Huang et al., 2002; Mulo et al., 2009; Hess et al., 2014; Maekawa et al., 2015). Therefore, the transcription of D1-encoding *MaPsbA3* was determined. Compared to the control, *MaPsbA3* was upregulated to 7.47-fold (*p* < 0.001) in N-depletion cells and 2.57-fold (*p* < 0.01) in SHX-treated cells. While NaCl and H_2_O_2_ stresses markedly reduced *MaPsbA3* transcripts to 0.47-fold (*p* < 0.05) and 0.72-fold (*p* < 0.01), respectively (Figure 7C), pretreatment with N depletion or SHX released the inhibition by NaCl or H_2_O_2_, as the transcription of *MaPsbA3* was recovered to 1.69-fold (*p* < 0.05) (N^–^ + NaCl), 2.19-fold (*p* < 0.05) (SHX + NaCl), 1.34-fold (*p* < 0.05) (N^–^ + H_2_O_2_), and 1.28-fold (*p* < 0.05) (SHX + H_2_O_2_). SR upregulated the transcription of *MaPsbA3* and improved the turnover of D1 and, thus, enhanced photorepair.

## Discussion

### N-Depletion-Induced SR Confers Stress Tolerance to *M. aeruginosa*

The present study provides evidence that SR triggered by N depletion or SHX conferred *M. aeruginosa* cells enhanced tolerance to environmental stress. The initiation of SR exerted multiple protective effects on *M. aeruginosa* cells to minimally disturb cellular physiology and prolong cell survival in stress conditions. Several factors contributed to the stress resistance, e.g., maintaining membrane integrity (Figure 3), segregating cells from harmful microenvironment by forming cell clusters which may be indicative of colonies rich in EPS (Figure 4), improving photosynthetic performance (Figure 5) by upregulating D1 synthesis (Figure 7C), and reducing endogenous ROS production (Figures 5, 6) by activating ROS-scavenging enzymes (Figure 7). In the state of SR, cellular morphology was changed markedly from a single-cell form to a multicellular colony (Figure 4). The colony of *M. aeruginosa* was wrapped by a cloud of EPS matrix, which functioned as a barrier between cells and the external environment to segregate cells from hazardous substances. Under the protection of EPS, *M. aeruginosa* cells were not bleached and lysed by high concentrations of NaCl (Figure 3). In addition, EPS can also contribute to the formation of *M. aeruginosa* aggregates and the final development of bloom from aggregates (Xu et al., 2014). Furthermore, SR-induced cells covered by EPS were less susceptible to damage by NaCl and H_2_O_2_ and therefore exhibited a higher *F*_*v*_/*F*_*m*_ ratio (Figure 5). SR also strengthened D1 biosynthesis to further enhance photosynthesis (Figure 7). Higher photosynthetic performance reduced electron leak during electron transfer reaction in the chloroplast and subsequently decreased ROS production (Figures 5, 6). Besides, SR enhanced ROS elimination by upregulating ROS-scavenging enzymes (Figure 7). Moreover, the robust repair of photodamage through upregulating *MaPsbA3* may mitigate the toxic effect of ROS (Figure 7). Therefore, a combination of reduced ROS production with enhanced ROS scavenging ability enabled cells to safeguard from ROS damage during stressing. Since overaccumulation of ROS can lead to irreversible damage and even may ultimately kill cells by reacting with a large number of biomolecules (Mittler, 2002), we assume that reducing the level of ROS as much as possible is one adaptation of SR-induced cells to resist stress. Similar results were observed in *P. aeruginosa*. SR activated by N depletion enhanced tolerance of *P. aeruginosa* to antibiotics by increasing the activities of SOD and catalase (Nguyen et al., 2011). N depletion may endow cells with tolerance to acute environmental stress by initiating SR.

### The Role of SR in Cyanobacterial Blooms

In the past decade, aquatic scientists advocated that besides P input reducing, the need to reduce N input should also be considered to control cyanobacterial blooms (Conley et al., 2009; Paerl et al., 2011). However, evidences show that N limitation cannot effectively control cyanobacterial blooms; instead, N limitation induces the bloom of N-fixation cyanobacteria (Conley et al., 2009; Paerl et al., 2011). For example, controlling N inputs actually aggravated the dominance of N-fixing cyanobacteria, and then new N fixed by N-fixing cyanobacteria replenished the N pool to promote the growth of non-N-fixing cyanobacteria in Lake 227 in northwestern Ontario, Canada (Schindler et al., 2008). In Lake Mendota (Wisconsin, United States), a decline in dissolved inorganic nitrogen resulted in the dominance of N-fixing cyanobacteria (e.g., *Aphanizomenon*) (Beversdorf et al., 2013). Although the effectiveness of controlling N to eliminate eutrophication had been observed in macroscale, the cellular-level mechanism underlying the ecological event is still poorly understood. The present study and several previous reports imply that SR may be involved in this process.

It seems that SR is widely distributed in cyanobacteria since bioinformatics analysis found that many cyanobacteria possess RSH analogs (Atkinson et al., 2011). N limitation probably triggers SR in cyanobacteria to regulate physiological activities. SR was proven to regulate stable RNA synthesis (Smith and Carr, 1977); and N depletion could induce ppGpp synthesis in *Anacystis nidulans* (Friga et al., 1981). In *Anabaena cylindrical*, SR was also triggered by N depletion (Akinyanju and Smith, 1979, 1987). Non-N-fixing and N-fixing cyanobacteria have developed different strategies to cope with N depletion, both involved SR. In non-N-fixing cyanobacteria, SR regulates cells to divert cellular resource from rapid growth to survival in natural water bodies, in which multiple stresses often occur simultaneously. For example, in this study, *M. aeruginosa* SR upregulated stress-related genes in response to N depletion. *Synechococcus* sp. differentiated to non-pigmented resting cells, known as chlorosis, to survive prolonged periods of N depletion (Sauer et al., 2001; Klotz et al., 2015). The formation of chlorosis under N depletion involved initiation of SR. In the chlorosis state, cells could live for several months under N depletion without loss of cell viability, and growth could be reinitiated immediately once N was supplied. SR also regulated the acclimation of *S. elongates* to darkness.

In N-fixing cyanobacteria, SR is involved in biological N fixation. N limitation encourages replacement of non-N-fixing with N-fixing cyanobacteria, such as *Anabaena* sp. and *Aphanizomenon* sp. (Smith, 1983, 1990; Schindler et al., 2008). These N-fixing cyanobacteria finally bring new N into the waterbody. It is reported that biological N fixation by cyanobacteria could reach 130–400 KT and support 45% of N accumulation in sediment in the Gotland Sea (Struck et al., 2004). It is found that N fixation was proportional to heterocyst numbers in filamentous cyanobacteria (Findlay et al., 1994) and SR was involved in heterocyst development (Wood and Haselkorn, 1980; Zhang et al., 2013). A representative example is *Anabaena* sp., in which SR was triggered by N depletion; SR then activated the formation of the N-fixing heterocyst, while the mutant loss of SR could not form the heterocyst (Zhang et al., 2013). Input of new N by N-fixing cyanobacteria may stimulate subsequent blooms of non-N-fixing cyanobacteria and other benthic algae. When new fixed N arrived in the waterbody, the resting non-N-fixing cells resumed to grow rapidly and alternatively became dominant (Paerl and Otten, 2016; Pérez et al., 2017; Chia et al., 2018).

Since SR is conserved in nearly all bacteria and N depletion triggers SR in many cyanobacteria, we speculate that SR may be involved in the succession of cyanobacterial blooms during N limitation in marine environments and some trophic lakes. SR protects cyanobacteria from environmental stress, which allows the cells to survive and sustain non-N-fixing cyanobacterial population to prepare for the next bloom, when new N sources is supplied by N-fixing cyanobacteria. That is, SR functions to (1) steady non-N-fixing cyanobacterial population and (2) promote new N supply by N-fixing cyanobacteria. Therefore, our work provided evidence at the cellular level that N limitation is ineffective to control cyanobacterial blooms. The alternated dominance between N-fixing and non-N-fixing cyanobacteria may be coordinated by SR.

## Data Availability Statement

All datasets generated for this study are included in the article/Supplementary Material.

## Author Contributions

HJ, YL, and ZC designed the research and wrote the manuscript. HJ, YL, and KY performed the experiments. HJ, YL, and JZ analyzed the data. All authors contributed to the article and approved the submitted version.

## Conflict of Interest

The authors declare that the research was conducted in the absence of any commercial or financial relationships that could be construed as a potential conflict of interest.

## Footnotes

1 http://blast.ncbi.nlm.nih.gov/

2 https://www.ncbi.nlm.nih.gov/Structure/cdd/wrpsb.cgi

3 https://swissmodel.expasy.org/
