# Supplementary material for: Stringent Response Regulates Stress Resistance in Cyanobacterium Microcystis aeruginosa
Source: Front Microbiol. 2020 Nov 12;11:511801. doi: 10.3389/fmicb.2020.511801 (PMC7688982; doi:10.3389/fmicb.2020.511801)
Supplement: Supplementary file 1 [file Data_Sheet_1.docx]

Supplementary Material

# Supplementary Tables

**Supplementary Table S1.** Primers used in this study.

| **Manipulation** | **Primer** | **Sequence (5’ to 3’)** |
| --- | --- | --- |
| Gene isolation | eMaRSH F | TAA***GGATCC***ATGAACGCCATCACTGC |
|  | eMaRSH R | TCT***AAGCTT***CTCTCGATCGCTTTTTACCTG |
| qRT-PCR | qMaRSH F | ATTTTCGCCGAATGTTCCTGG |
|  | qMaRSH R | TTGCTGCTGTTGCGGGTTGA |
|  | qMaSOD F | CCGTTATCGAGAACCAACCAAGCC |
|  | qMaSOD R | TTTTCAACAATGCCGCCCAAG |
|  | qMaPOD F | AACGAGATACTGCGGGTGATTGACT |
|  | qMaPOD R | AACTCCTTTGGGGAATTTGCTACG |
|  | qMaGR F | ACCCGTAACCTTCCGCATCCT |
|  | qMaGR R | TGGTGGTGGTTCTGGTGGTATTG |
|  | qMaGAPX F | CCACCTGTTCCACCTCCCATTTAC |
|  | qMaGAPX R | CATTCTTGATCTTTTGCCCACTCG |
|  | qMa16S F | ACTGCTAATACCCGATATGCCGC |
|  | qMa16S R | AATCCAAAGACCTTCCTCCCTCA |
|  | qMaPsbA3 F | TCTTTCAACAATAGCCGCTCTCTG |
|  | qMaPsbA3 R | TTGCGCTCGTGCATTACTTCC |

Restriction sites are shown in bold italics.

**Supplementary Table S2.** Sequences used for sequence alignment

| **Enzyme** | **Species** | **Accession no.** |
| --- | --- | --- |
| MaRSH | *Microcystis aeruginosa* | KU744003 |
| CeRelA | *Crinalium epipsammum* | AFZ13075 |
| TeRelA | *Thermosynechococcus elongates* | BAC08136 |
| PmSpoT | *Prochlorococcus marinus* | CAE22285 |
| AtRSH | *Arabidopsis thaliana* | AAF37281 |
| NtRSH | *Nicotiana tabacum* | AAQ23899 |
| SbRSH | *Sorghum bicolor* | XP_002467885 |
| SeRel | *Streptococcus equisimilis* | 1VJ7_B |
| BsRelA | *Bacillus subtilis* | AAC46041 |
| EcSpoT | *Escherichia coli* | AAB00160 |
| EcRelA | *Escherichia coli* | AAA03237 |
| CjSpoT | *Campylobacter jejuni* | CAL35387 |
| MtRelA | *Mycobacterium tuberculosis* | AJK62606 |
| HpRSH | *Haematococcus pluvialis* | KU744004 |
| CrRSH | *Chlamydomonas reinhardtii* | BAB91333 |
